# Psychische Gesundheit am Arbeitsplatz – Welche Rolle spielen sozioökonomische, geschlechterspezifische und migrationsbedingte Ungleichheiten?

**DOI:** 10.1007/s00103-024-03902-9

**Published:** 2024-06-19

**Authors:** Regina Herold, Manuel Feißt, Eva Morawa, Sinja Hondong, Eva Rothermund, Tamara Waldmann, Meike Heming, Jeannette Weber, Nicole R. Hander, Nadine Mulfinger, Christoph Kröger, Yesim Erim

**Affiliations:** 1 Psychosomatische und Psychotherapeutische Abteilung, Universitätsklinikum Erlangen, Friedrich-Alexander-Universität Erlangen-Nürnberg (FAU), Schwabachanlage 6, 91054 Erlangen, Deutschland; 2 Institut für Medizinische Biometrie, Universitätsklinikum Heidelberg, Heidelberg, Deutschland; 3 Klinik für Psychosomatische Medizin und Psychotherapie, Universitätsklinikum Ulm, Ulm, Deutschland; 4 Klinik für Psychiatrie und Psychotherapie II der Universität Ulm am Bezirkskrankenhaus Günzburg, Günzburg, Deutschland; 5 Institut für Arbeits‑, Sozial- und Umweltmedizin, Centre of Health and Society, Medizinische Fakultät und Universitätsklinikum Düsseldorf, Heinrich-Heine-Universität Düsseldorf, Düsseldorf, Deutschland; 6 Institut für Psychologie, Abteilung Klinische Psychologie und Psychotherapie, Universität Hildesheim, Hildesheim, Deutschland

**Keywords:** Arbeitsbedingungen, Geschlechterunterschiede, Migration, Depression, Akkulturation, Working conditions, Gender differences, Migration, Depression, Acculturation

## Abstract

**Hintergrund:**

Risikofaktoren für die psychische Gesundheit sind häufig in sozioökonomischen, geschlechter- und migrationsspezifischen Ungleichheiten begründet. Diese sowie die Ausprägung der Depressivität, Ängstlichkeit und Somatisierung von Beschäftigten wurden in der vorliegenden Studie untersucht.

**Methoden:**

Im Rahmen der friaa-Studie („Frühe Intervention am Arbeitsplatz“) wurden psychisch belastete Beschäftigte an 5 Standorten in Deutschland zu soziodemografischen, arbeits-, migrations- und gesundheitsbezogenen Inhalten befragt. Mittels Regressionsanalysen wurde der Zusammenhang dieser Faktoren mit Depressivität (Patient-Health-Questionnaire-9, PHQ-9), Ängstlichkeit (Generalized Anxiety Disorder-2, GAD-2) und Somatisierung (Somatic Symptom Scale-8, SSS-8) an der gesamten Stichprobe sowie bei Personen mit Migrationshintergrund (MH) untersucht. Bei Letzteren wurden zusätzlich die Akkulturation (Frankfurter Akkulturationsskala, FRAKK) und das Belastungsempfinden gegenüber Migrationsanforderungen (Demands of Immigration Scale, DIS) berücksichtigt.

**Ergebnisse:**

Die 550 Beschäftigten (12 % mit MH) wiesen im Durchschnitt eine klinisch relevante Depressivität (M = 13,0, SD = 5,1) (PHQ-9 ≥ 10), Ängstlichkeit (M = 3,5, SD = 1,7) (GAD ≥ 3) sowie Somatisierung (M = 13,0, SD = 5,8) (SSS-8 ≥ 12) auf. Das weibliche Geschlecht war mit höheren Werten für Ängstlichkeit und Somatisierung assoziiert. Höheres Alter und Nachtschichtarbeit hingen mit stärkerer Somatisierung zusammen.

**Diskussion:**

Die Ergebnisse bestätigen die hohe psychische Belastung der Beschäftigten in dieser Stichprobe aus Deutschland. Um deren psychische Gesundheit aufrechtzuerhalten, sollten Unterstützungsmaßnahmen insbesondere für vulnerable Gruppen wie Frauen, ältere Beschäftigte und Nachtschichtarbeitende angeboten werden.

## Abstract (en)

**Background:**

Risk factors for mental health can be found in socio-economic-, gender- and migration-specific inequalities. These factors and the extent of depression, anxiety, and somatization among employees were examined in the present study.

**Methods:**

As part of the Early Intervention in the Workplace Study (friaa), mentally burdened employees at five locations in Germany were surveyed on socio-demographic-, work-, migration-, and health-related content. Regression analyses were used to examine the relationship between these factors and depression (Patient-Health-Questionnaire-9, PHQ-9), anxiety (Generalized Anxiety Disorder-2, GAD-2), and somatization (Somatic Symptom Scale-8, SSS-8) in the entire sample and in people with migration background (MB). For the latter, acculturation (Frankfurt Acculturation Scale, FRACC) and the perception of burden in terms of demands of immigration (Demands of Immigration Scale, DIS) were also taken into account.

**Results:**

On average, the 550 employees (12% with MB) showed clinically relevant depression (M = 13.0, SD = 5.1) (PHQ-9 ≥ 10), anxiety (M = 3.5, SD = 1.7) (GAD ≥ 3) and somatization (M = 13.0, SD = 5.8) (SSS-8 ≥ 12). Female gender was associated with higher anxiety and somatization. Older age and night shift work were associated with higher somatization.

**Discussion:**

The results confirm the high level of mental burden among this sample of employees in Germany. In order to maintain their mental health, support measures should be offered, especially for vulnerable groups such as women, older employees, and night shift workers.

## Hintergrund

Eine repräsentative Untersuchung aus dem Jahr 2016 ergab, dass 10 % aller Beschäftigten in Deutschland unter Burnout-Symptomen litten [1]. Laut einer aktuellen Untersuchung der Techniker Krankenkasse gaben fast 40 % aller befragten 1098 Geschäftsführenden, Gesundheitsverantwortlichen und Personaler:innen an, dass psychische Probleme wie Burnout, Überforderungsgefühle oder Depression unter Beschäftigten in ihren Unternehmen eine bedeutsame Relevanz besäßen [2]. Eine Analyse der finanziellen Konsequenzen zeigte, dass psychische Erkrankungen mit ca. 18 % den zweithäufigsten Grund für Arbeitsunfähigkeitstage in Deutschland darstellen, womit enorme finanzielle Einbußen einhergehen [3]. Die geschilderten Daten verdeutlichen eine hohe gesellschaftliche und politische Relevanz der psychischen Belastungen bei Berufstätigen.

Ungleichheiten führen in benachteiligten Subgruppen zu einer schlechteren Gesundheit [4] und höherer Sterblichkeit [5]. Zu den Determinanten zählen Geschlecht, Alter, sozioökonomischer Status, Migrationserfahrung und Arbeitsbedingungen [6]. Frauen sind häufiger psychisch belastet als Männer [7, 8]. Kinder und Jugendliche aus Familien mit einem geringen sozioökonomischen Status wiesen ein 2‑ bis 3‑fach erhöhtes Risiko auf, unter einer schlechteren psychischen Gesundheit zu leiden [9]. Unter Erwachsenen wurde deutlich, dass ein geringer sozioökonomischer Status mit psychischen sowie körperlichen Erkrankungen zusammenhing [10]. Seitens der Arbeitsbedingungen führten hohe quantitative Anforderungen (z. B. schnelles Arbeiten) unter Beschäftigten häufiger zu Burnout- und Depressionssymptomen [1]. Auch ein Migrationshintergrund (MH) wies einen Zusammenhang mit höherer psychischer Belastung auf. Migrant:innen litten häufiger unter einer beeinträchtigten (mentalen) Gesundheit als Personen ohne MH [11, 12]. Dies liegt u. a. an einer Benachteiligung im Arbeitsleben im Vergleich zu Personen ohne MH [13].

Eine gleichzeitige Berücksichtigung verschiedener Determinanten führt zu einer präzisen Identifikation von Ungleichheiten, einer gezielten Entwicklung von Interventionsstrategien und somit einem effektiven Ansatz, Benachteiligungen in der mentalen Gesundheit zu minimieren. Aus diesem Grund beschäftigt sich die vorliegende Arbeit mit sozioökonomischen, geschlechter- und migrationsbedingten Ungleichheiten bei psychisch belasteten Beschäftigten, welche Bedarf an einer frühen psychotherapeutischen Intervention am Arbeitsplatz anmeldeten. Durch das Befragen psychisch belasteter Beschäftigter können spezifische protektive sowie Risikofaktoren ausgemacht werden, wodurch (z. B. in psychotherapeutischen Interventionen) effektiv auf diese Subpopulation und deren Bedürfnisse eingegangen werden kann. Zu diesem Zweck wurde untersucht, ob soziodemografische Variablen wie Alter, Geschlecht und Familienstand, aber auch arbeitsbedingte Parameter wie wöchentliche Arbeitsstunden, Schichtarbeit mit und ohne Nachtschicht und das Bekleiden einer Führungsposition sowie migrationsspezifische Faktoren wie der MH, der Grad der soziokulturellen Akkulturation (Orientierung an der Herkunfts- und Aufnahmekultur) und das Belastungsempfinden bezüglich Migrationsanforderungen einen Zusammenhang mit psychischen Problemen in Form von Depressivität, Ängstlichkeit und Somatisierung aufweisen.

## Methoden

### Studienpopulation, Studiendesign und Setting

Für die vorliegende Analyse wurden Daten der Baseline-Messung einer BMBF-geförderten randomisiert-kontrollierten Studie genutzt, die das Ziel verfolgte, psychisch belasteten Beschäftigten eine frühe psychotherapeutische Intervention („Frühe Intervention am Arbeitsplatz“, friaa) zu ermöglichen, ehe sich krankheitswertige Störungsbilder manifestieren. Die Teilnehmer:innen wurden u. a. durch Betriebsärzt:innen und teilweise über soziale Medien und lokale Zeitungen aus Groß-, mittelständischen und Klein(st)unternehmen aus 5 Studienzentren (Ulm, Düsseldorf, Teltow, Hildesheim und Erlangen) rekrutiert. Die Teilnehmenden mussten mindestens 18 Jahre alt sein, über ausreichende Deutschkenntnisse verfügen, mindestens 15 Stunden in der Woche arbeiten sowie entweder über die Diagnose einer unipolaren depressiven, einer Ängstlichkeits-, einer stressbezogenen oder somatoformen Störung oder einer nichtorganischen Schlafstörung verfügen oder subklinische Symptome psychosomatischer Störungen aufweisen. Nicht eingeschlossen wurden Personen, die die Diagnose eines Substanzmissbrauchs, einer Schizophrenie, einer Psychose oder einer organischen psychiatrischen Störung erfüllten oder sich in einem instabilen somatischen Zustand befanden (z. B. Krebserkrankung). Auch durften die Personen aktuell keiner psychotherapeutischen Intervention beiwohnen oder einen Rentenantrag gestellt haben [14]. Bei Eignung unterschrieben die Proband:innen eine schriftliche Einverständniserklärung zur Teilnahme und füllten vor Interventionsbeginn einen Online-Fragebogen in REDCap (Research Electronic Data Capture; [15, 16]) aus. Inhalte des Fragebogens waren allgemeine soziodemografische sowie arbeitsbezogene Fragen, Fragen zur körperlichen und psychischen Gesundheit, zu einer vorangegangenen Psychotherapie, migrationsspezifische Fragen und Fragen zu Klinikaufenthalten und anderen Behandlungsarten. Die die vorliegende Arbeit nicht betreffenden Ergebnisse werden in anderen Publikationen behandelt.

### Messinstrumente (Selbstbeurteilungsfragebögen)

#### Soziodemografische Daten.

Die erfragten soziodemografischen Daten umfassten Geschlecht, Alter und Familienstand.

#### Migrationsspezifische Daten.

Der MH wurde erhoben, indem nach der Definition des Mikrozensus erfragt wurde, ob man selbst die deutsche Staatsangehörigkeit nicht seit Geburt besitzt (erste Migrationsgeneration (MG)) und ob mindestens ein Elternteil die deutsche Staatsangehörigkeit nicht von Geburt an besitzt (zweite MG; [17]). Personen mit MH wurden hinsichtlich ihrer Deutschkenntnisse, ihres aktuellen Aufenthaltsstatus, ihres Migrationsmotivs und ihrer Aufenthaltsdauer in Deutschland befragt.

Bei Personen mit MH wurde mithilfe von 20 Items der deutschen validierten Version der Frankfurter Akkulturationsskala (FRAKK) der individuelle Grad der Akkulturation erfragt. Diese gibt unter Benutzung des Summenwertes der jeweiligen Subskala (Wertebereich von 0–60) an, in welchem Maß sich eine Person mit MH an der Aufnahme- und an der Herkunftskultur orientiert. Ein höherer Wert spricht für eine jeweils höhere Orientierung [18]. In der vorliegenden Stichprobe lässt sich die interne Konsistenz der Subskala „Orientierung an der Aufnahmekultur“ mit Cronbachs α = 0,78 als akzeptabel und die der Subskala „Orientierung an Herkunftskultur“ mit Cronbachs α = 0,87 als hoch beschreiben.

Die validierte deutsche Version der Demands of Immigration Scale (DIS) wurde eingesetzt, um bei Personen mit MH den Grad der Belastung zu erfassen, der mit Einwanderungsanforderungen verbunden ist. Sie besteht aus 23 Items, die auf einer Skala von 0 bis 3 beantwortet werden, und erfragt die wahrgenommene Belastung in 6 Subskalen: Verlust, Neuheit, berufliche Passung, Sprache, Diskriminierung, sich im Aufnahmeland nicht zuhause fühlen. Ein höherer Summenwert bedeutet eine höhere Belastung [19]. Die DIS wies mit Cronbachs α = 0,89 in der vorliegenden Studie eine hohe interne Konsistenz auf.

#### Arbeitsbezogene Daten.

Es wurden die derzeitige Erwerbssituation, wöchentliche Arbeitsstunden und Arbeitstage, Schichtarbeit mit und ohne Nachtschicht, das Alter beim ersten Job, die Berufsgruppe und die Betriebsgröße erhoben. Personen mit Führungsposition gaben die Mitarbeitendenanzahl an, für die sie Verantwortung trugen.

#### Gesundheitsbezogene Daten.

Es wurde erfragt, ob innerhalb der letzten 6 Monate aus gesundheitlichen Gründen der Arbeit ferngeblieben wurde, sowie die durchschnittliche Anzahl an Krankheitstagen.

Zur Erfassung der Depressivität kam der Patient-Health-Questionnaire‑9 (PHQ-9), bestehend aus 9 Items in der validierten deutschen Fassung zur Anwendung. Ab einem Summenwert von ≥ 10 kann von einer klinisch relevanten depressiven Symptomatik ausgegangen werden (Wertebereich von 0–27). Er umfasst Fragen wie: „Wie oft fühlten Sie sich im Verlauf der letzten zwei Wochen durch wenig Interesse oder Freude an Ihren Tätigkeiten beeinträchtigt?“ [20]. Die interne Konsistenz des PHQ‑9 ist mit Cronbachs α = 0,80 in der vorliegenden Stichprobe als hoch einzuschätzen.

Ängstlichkeit wurde mithilfe des Fragebogens Generalized Anxiety Disorder‑2 (GAD-2), welcher aus 2 Items besteht, in der validierten deutschen Version genutzt. Ein Summenwert von ≥ 3 deutet auf eine klinisch relevante Ängstlichkeitssymptomatik hin (Wertebereich von 0–6). Er beinhaltet Fragen wie: „Wie oft fühlten Sie sich im Verlauf der letzten zwei Wochen durch Nervosität, Ängstlichkeit oder Anspannung beeinträchtigt?“ [21]. Die interne Konsistenz des GAD‑2 lässt sich in der vorliegenden Studie als akzeptabel einstufen (Cronbachs α = 0,75).

Die Somatic Symptom Scale‑8 (SSS-8), bestehend aus 8 Items, wurde in der validierten deutschen Fassung genutzt, um somatische bzw. körperliche Symptome zu erfassen. Ein Summenwert von ≥ 12 deutet auf klinisch relevante Somatisierungssymptome hin (Wertebereich von 0–32). Eine Beispielfrage lautet: „Wie stark fühlten Sie sich im Verlauf der letzten sieben Tage durch Bauchschmerzen oder Verdauungsbeschwerden beeinträchtigt?“ [22]. Die interne Konsistenz des SSS‑8 lässt sich in der vorliegenden Stichprobe als akzeptabel interpretieren (Cronbachs α = 0,74).

### Statistische Analysen

Die statistischen Analysen wurden mit R‑4.2.0 durchgeführt. Aufgrund des explorativen Charakters der Analysen wurden fehlende Werte nicht imputiert. Zur Beschreibung der allgemeinen Soziodemografie und der migrations-, arbeits- und gesundheitsbezogenen Angaben wurden relative und absolute Häufigkeiten von Summen- und Mittelwerten (M) und deren Standardabweichungen (SD) genutzt. Vergleiche zwischen Personen mit und ohne MH und zwischen Personen der ersten und zweiten MG wurden mithilfe von exakten Fisher-Tests, Boschloos Tests sowie 2‑seitigen (Welch-)t-Tests durchgeführt. Um den Zusammenhang zwischen den wichtigsten sozioökonomischen, geschlechter- und migrationsbedingten Daten und der psychischen Gesundheit (Depressivität, Ängstlichkeit, Somatisierung) zu untersuchen, wurde für die gesamte Stichprobe für jedes Gesundheitsoutcome jeweils eine multivariable lineare Regressionsanalyse mit den unabhängigen Faktoren Geschlecht (weiblich vs. männlich/divers), Alter, Familienstand (keine Partnerschaft vs. Partnerschaft), wöchentliche Arbeitsstunden, Schichtarbeit mit Nachtschicht (vs. keine Schichtarbeit), Schichtarbeit ohne Nachtschicht (vs. keine Schichtarbeit), Führungsposition (Ja vs. Nein) und MH (Nein vs. Ja) berechnet. Um Zusammenhänge vor allem zwischen migrationsspezifischen Angaben und der mentalen Gesundheit genauer zu beleuchten, wurden zudem für die Subgruppe der Personen mit MH 3 multivariable lineare Regressionsanalysen mit den Faktoren Geschlecht, Alter, Orientierung an der Aufnahmekultur, Orientierung an der Herkunftskultur, wöchentliche Arbeitsstunden, Schichtarbeit mit Nachtschicht, Schichtarbeit ohne Nachtschicht und Führungsposition durchgeführt. Unter Berücksichtigung des standardisierten Regressionskoeffizienten werden sowohl statistisch signifikante als auch klinisch relevante Ergebnisse berichtet (β ≥ 0,1 [23]).

Für die Prüfung der statistischen Signifikanz wurde ein Alpha-Fehler-Niveau von 0,05 (2-seitig) verwendet. Aufgrund des explorativen Charakters der Auswertung ist diese rein deskriptiv zu interpretieren.

## Ergebnisse

### Allgemeine Soziodemografie

Soziodemografische Informationen finden sich in Tab. 1. Über die Hälfte der Proband:innen war weiblich (54,7 %, *n* = 301; Tab. 1). Das Durchschnittsalter betrug 46 Jahre (SD = 11,0). Die Mehrheit der Proband:innen befand sich in einer festen Partnerschaft oder war verheiratet (60,4 %, *n* = 332).

**Tab. 1:** Soziodemografische und arbeitsbezogene Daten der gesamten Stichprobe

	Gesamtstichprobe, *n* = 550 (100 %)	Personen ohne MH^a^, *n* = 482 (88 %)	Personen mit MH^a^, *n* = 68 (12 %)	Unterschied zwischen Personen mit und ohne MH^ab^, *p*-Wert	Personen der 1. Migrationsgeneration, *n* = 36 (52,9 %^c^)	Personen der 2. Migrationsgeneration, *n* = 32 (47,1 %^c^)	Unterschied zwischen Personen der 1. und 2. Migrationsgeneration^b^, *p*-Wert
**Geschlecht, *n* (%)**	–	–	–	0,293	–	–	0,367
Weiblich	301 (55)	269 (56)	32 (47)		15 (42)	17 (53)	
Männlich	247 (45)	211 (44)	36 (53)		21 (58)	15 (47)	
Divers	1 (0)	1 (0)	0 (0)		0 (0)	0 (0)	
Fehlend	1 (0)	1 (0)	0 (0)		0 (0)	0 (0)	
**Alter, M (SD; Min.–Max.)**	46 (11,0) (21–64)	46 (11,0) (21–64)	42 (11,0) (21–62)	**0,005**	44 (10,7)	32 (11,2)	0,336
Fehlend, *n* (%)	1 (0)	1 (0)	0 (0)		0 (0)	0 (0)	
**Familienstand, *n* (%)**	–	–	–	0,858	–	–	0,231
Verheiratet/in fester Partnerschaft	332 (61)	289 (60)	43 (63)		25 (69)	18 (56)	
Ledig	144 (26)	127 (26)	17 (25)		9 (25)	8 (25)	
Geschieden	63 (12)	55 (11)	8 (12)		2 (6)	6 (19)	
Verwitwet	10 (2)	10 (2)	0 (0)		0 (0)	0 (0)	
Fehlend	1 (0)	1 (0)	0 (0)		0 (0)	0 (0)	
**Erwerbssituation, *n* (%)**	–	–	–	0,272	–	–	0,430
Vollzeit	402 (73)	347 (72)	55 (81)		31 (86)	24 (75)	
Sonstiges^d^	147 (27)	134 (28)	13 (19)		5 (14)	8 (25)	
Fehlend	1 (0)	1 (0)	0 (0)		0 (0)	0 (0)	
**Wochenarbeitsstunden, M (SD; Min.–Max.)**	37 (7,1) (7–75)	37 (7,1) (7–60)	38 (7,4) (20–75)	0,297	38 (8,8) (20–75)	37 (5,3) (20–50)	0,630
Fehlend, *n* (%)	4 (1)	3 (1)	1 (1)		0 (0)	1 (3)	
**Wochenarbeitstage, M (SD; Min.–Max.)**	5 (0,6) (0–7)	5 (0,6) (3–7)	5 (0,6) (0–7)	0,073	5 (0,6) (4–7)	5 (0,5) (3–6)	0,075
Fehlend, *n* (%)	1 (0)	1 (0)	0 (0)		0 (0)	0 (0)	
**Schichtarbeit, *n* (%)**	–	–	–	0,554	–	–	0,793
Nein	475 (87)	418 (87)	57 (84)		29 (81)	28 (88)	
Ja, mit Nachtschicht	40 (7)	35 (7)	5 (7)		3 (8)	2 (6)	
Ja, ohne Nachtschicht	34 (6)	28 (6)	6 (9)		4 (11)	2 (6)	
Fehlend	1 (0)	1 (0)	0 (0)		0 (0)	0 (0)	
**Alter beim ersten Job, M (SD)**	20 (4,9)	20 (4,8)	19 (5,3)	0,873	19 (3,5)	20 (6,8)	0,261
Fehlend	2 (0)	2 (0)	0 (0)		0 (0)	0 (0)	
**Berufsgruppe, *n* (%)**	–	–	–	NA^e^	–	–	0,257
Ungelernte:r Arbeiter:in	18 (3)	12 (2)	6 (9)		5 (14)	1 (3)	
Gelernte:r Arbeiter:in/Facharbeiter:in	104 (19)	86 (18)	18 (27)		10 (28)	8 (25)	
Vorarbeiter:in	3 (1)	3 (1)	0 (0)		0 (0)	0 (0)	
Selbstständige:r	3 (1)	3 (1)	0 (0)		0 (0)	0 (0)	
Ausführende:r Angestellte:r/Beamte:r	30 (5)	27 (6)	3 (4)		3 (8)	0 (0)	
Mittlerer Angestellte:r/Beamte:r	246 (45)	220 (46)	26 (38)		12 (33)	14 (44)	
Leitende:r Angestellte:r/Beamte:r	107 (19)	96 (20)	11 (16)		5 (14)	6 (19)	
Sonstiges	38 (7)	34 (7)	4 (6)		1 (3)	3 (9)	
Fehlend	1 (0)	1 (0)	0 (0)		0 (0)	0 (0)	
**Führungsposition, *n* (%)**	–	–	–	0,112	–	–	0,270
Nein	433 (79)	374 (78)	59 (87)		33 (92)	26 (81)	
Ja	116 (21)	107 (22)	9 (13)		3 (8)	6 (19)	
Fehlend	1 (0)	1 (0)	0 (0)		0 (0)	0 (0)	
**Anzahl der Mitarbeitenden, für die man Verantwortung trägt**^f^**, M (SD)**	40 (10,0)	26 (41,7)	8 (6,1)	**<** **0,001**	5 (5,0)	10 (6,3)	0,266
Fehlend	9 (2)	0 (0)	9 (13)		3 (8)	6 (19)	
**Betriebsgröße, *n* (%)**	–	–	–	**0,007**	–	–	0,479
Großbetrieb^g^	439 (80)	391 (81)	48 (71)		24 (67)	24 (77)	
Mittelständisches Unternehmen^h^	60 (11)	46 (10)	14 (21)		8 (22)	6 (19)	
Klein(st)unternehmen/Einmannbetrieb^i^	49 (9)	44 (9)	5 (7)		4 (11)	1 (3)	
Fehlend	2 (0)	1 (0)	1 (1)		0 (0)	1 (3)	

^a^Migrationshintergrund

^b^Berechnet mit exakten Fisher-Tests, Boschloos Tests oder 2‑seitigen Welch-t-Tests, signifikante Unterschiede werden fett hervorgehoben

^c^Prozentangabe gemessen an allen Personen mit Migrationshintergrund

^d^Diese Kategorie beinhaltet: Teilzeit, freiwillig (unentgeltlich) beschäftigt, geschützte Tätigkeit, nicht zutreffend

^e^Kein exakter Fisher-Test möglich

^f^Bezieht sich nur auf die Personen, die eine Führungsposition bejahten

^g^Ab 250 Mitarbeitenden

^h^50–249 Mitarbeitende

^i^1–49 Mitarbeitende

### Migrationsspezifische Daten

In der Stichprobe gaben 68 Personen (12,4 %) an, einen MH zu besitzen (Tab. 2). Von diesen zählten 52,9 % (*n* = 36) zur ersten MG.

**Tab. 2:** Migrationsspezifische Daten zu Personen mit Migrationshintergrund (erste und zweite Migrationsgeneration)

	Personen mit MH^a^, *n* = 68 (100 %)	Personen der 1. MG^b^, *n* = 36 (53 %)	Personen der 2. MG^b^, *n* = 32 (47 %)	Unterschied zwischen Personen der 1. und 2. MG^b^, *p*-Wert^c^
**Deutschkenntnisse, *n* (%)**	–	–	–	**<** **0,001**
Muttersprache	40 (59)	12 (33)	28 (88)	
Sehr gut	16 (24)	14 (39)	2 (6)	
Gut	6 (9)	5 (14)	1 (3)	
Mittelmäßig	5 (6)	4 (11)	1 (3)	
Schlecht	0 (0)	0 (0)	0 (0)	
Sehr schlecht	1 (2)	1 (3)	0 (0)	
**Aufenthaltsstatus, *n* (%)**	–	–	–	**<** **0,001**
Deutsche Staatsangehörigkeit	50 (74)	18 (50)	32 (100)	
Andere Staatsangehörigkeit	5 (7)	5 (14)	0 (0)	
Niederlassungserlaubnis oder Daueraufenthaltsrecht^d^	9 (13)	9 (25)	0 (0)	
Befristete Aufenthaltserlaubnis	2 (3)	2 (5)	0 (0)	
Duldung	0 (0)	0 (0)	0 (0)	
Uneingeschränktes Aufenthaltsrecht	2 (3)	2 (6)	0 (0)	
Touristenvisum	0 (0)	0 (0)	0 (0)	
**Migrationsmotiv, *n* (%)**	–	–	–	**0,003**
Seit Geburt in Deutschland	38 (58)	12 (34)	26 (84)	
Arbeit	6 (9)	4 (11)	2 (7)	
(Politisch oder religiös motivierte) Flucht	3 (5)	3 (9)	0 (0)	
Familiennachzug	6 (9)	5 (14)	1 (3)	
(Spät‑)Aussiedler	3 (5)	2 (6)	1 (3)	
Studium/Ausbildung	3 (5)	3 (9)	0 (0)	
Besserer Lebensstandard	4 (6)	3 (9)	1 (3)	
Sonstiges	3 (5)	3 (9)	0 (0)	
Fehlend	2 (3)	1 (3)	1 (0)	
**Aufenthaltsdauer**^e^**, M (SD; Min.–Max.)**	–	24 (11) (6–4)	–	–
Fehlend	–	13	–	
**FRAKK**^f^, **M (SD)**				
Orientierung an der Aufnahmekultur (AK)^g^	50 (8,2)	48 (8,4)	52 (7,7)	0,095
Fehlend	5 (7)	1 (3)	4 (13)	
Orientierung an der Herkunftskultur (HK)^g^	21 (14,0)	24 (12,0)	19 (17,0)	0,178
Fehlend	4 (6)	1 (3)	3 (9)	
**DIS**^h^**, M (SD)**				
Gesamtwert^i^	1,9 (2,0)	2,6 (2,3)	0,82 (1,0)	**<** **0,001**
Fehlend	14 (21)	4 (11)	10 (31)	
Verlust^i^	0,48 (0,66)	0,72 (0,76)	0,17 (0,31)	**<** **0,001**
Fehlend	10 (15)	3 (8)	7 (22)	
Neuheit^i^	0,21 (0,37)	0,30 (0,39)	0,09 (0,31)	**0,029**
Fehlend	10 (15)	3 (8)	7 (22)	
Arbeit^i^	0,25 (0,42)	0,33 (0,48)	0,15 (0,29)	0,091
Fehlend	11 (16)	3 (8)	8 (25)	
Sprache^i^	0,16 (0,50)	0,19 (0,40)	0,12 (0,61)	0,670
Fehlend	10 (15)	2 (6)	8 (25)	
Diskriminierung^i^	0,52 (0,67)	0,66 (0,77)	0,33 (0,44)	**0,049**
Fehlend	12 (18)	4 (11)	8 (25)	
Gefühl, nicht zuhause zu sein^i^	0,37 (0,60)	0,43 (0,65)	0,29 (0,52)	0,365
Fehlend	10 (15)	3 (8)	7 (22)	

^a^Migrationshintergrund

^b^Migrationsgeneration

^c^Berechnet mit exakten Fisher-Tests oder einseitigen t‑Tests, signifikante Unterschiede werden fett hervorgehoben

^d^Für Bürger der EU oder des Europäischen Wirtschaftsraums

^e^In Jahren, bezieht sich nur auf Personen der ersten Migrationsgeneration

^f^Frankfurter Akkulturationsskala

^g^Wertebereich von 0 bis 60

^h^Demands of Immigration Scale

^i^Wertebereich von 0 bis 3

Bezüglich des Akkulturationsgrades (FRAKK) erreichten die Personen mit MH einen Wert von 50 (SD = 8,2) auf der Skala „Orientierung an der Aufnahmekultur“ und einen Wert von 21 (SD = 14,0) auf der Skala „Orientierung an der Herkunftskultur“. Personen der ersten und zweiten MG (M = 52, SD = 7,7) unterschieden sich weder bezüglich der Orientierung an der Aufnahme- (M = 48, SD = 8,4 vs. M = 52, SD = 7,7, *p* = 0,095) noch der Herkunftskultur (M = 24, SD = 12 vs. M = 19, SD = 17, *p* = 0,178) signifikant.

In Bezug auf die DIS wiesen Personen mit MH einen Wert von 1,9 (SD = 2,0) auf. Personen der ersten MG gaben signifikant höhere Belastungen in Bezug auf Migrationsanforderungen an als Personen der zweiten MG (M = 2,6, SD = 2,3 vs. M = 0,82, SD = 1,0, *p* < 0,001). Signifikante Unterschiede zwischen Personen der ersten und zweiten MG sind vor allem in den Subskalen „Verlust“ (M = 0,72, SD = 0,76 vs. M = 0,17, SD = 0,31, *p* < 0,001), „Neuheit“ (M = 0,3, SD = 0,39 vs. M = 0,09, SD = 0,31, *p* = 0,029) und „Diskriminierung“ (M = 0,66, SD = 0,77 vs. M = 0,33, SD = 0,44, *p* = 0,049) erkennbar. Alle migrationsbezogenen Daten werden in Tab. 2 dargestellt.

### Arbeitsbezogene Angaben

Unter den Proband:innen waren 73,0 % (*n* = 402) in Vollzeit und 25,1 % (*n* = 138) in Teilzeit angestellt (Tab. 1). Die restlichen Teilnehmer:innen waren entweder freiwillig (unentgeltlich) beschäftigt, führten eine geschützte Tätigkeit aus oder die Antwortkategorien waren nicht zutreffend. Durchschnittlich arbeiteten die Proband:innen 37 Stunden (SD = 7,1) an 5 Tagen in der Woche (SD = 0,6). Etwa ein Fünftel hatte eine Führungsposition inne (21,1 %, *n* = 116). Die deutliche Mehrheit arbeitete nicht im Schichtdienst (86,4 %, *n* = 475). Unter den Schichtarbeitenden gab knapp über die Hälfte an, auch Nachtschichten zu haben (54,1 %, *n* = 40). In Tab. 1 sind alle arbeitsbezogenen Daten aufgeführt.

### Gesundheitsbezogene Angaben

Die deutliche Mehrheit der Proband:innen (74,4 %, *n* = 409) blieb innerhalb der letzten 6 Monate aufgrund gesundheitlicher Probleme der Arbeit fern (Tab. 3). Im Durchschnitt betraf das 22 Tage (SD = 33,0). Der Durchschnittswert auf der Depressionsskala lag in der gesamten Stichprobe bei M = 13,0 (SD = 5,1), auf der Ängstlichkeitsskala bei M = 3,5 (SD = 1,7) und auf der Somatisierungsskala bei M = 13,0 (SD = 5,8). Alle gesundheitsbezogenen Daten sind Tab. 3 zu entnehmen.

**Tab. 3:** Gesundheitsbezogene Daten der gesamten Stichprobe

	Gesamtstichprobe, *n* = 550 (100 %)	Personen ohne MH^a^, *n* = 482 (88 %)	Personen mit MH^a^, *n* = 68 (12 %)	Unterschied zwischen Personen mit und ohne MH, *p*-Wert^b^	Personen der 1. MG^c^, *n* = 36 (52,9 %^d^)	Personen der 2. MG^c^, *n* = 32 (47,1 %^c^)	Unterschied zwischen Personen der 1. und 2. MG^c^, *p*-Wert^b^
**Fernbleiben von der Arbeit aufgrund gesundheitlicher Gründe innerhalb der letzten 6 Monate, *n* (%)**	–	–	–	0,767	–	–	0,838
Nein	140 (26)	124 (26)	16 (24)		8 (22)	16 (24)	
Ja	409 (74)	357 (74)	52 (76)		28 (78)	52 (76)	
Fehlend	1 (0)	1 (0)	0 (0)		0 (0)	0 (0)	
**Anzahl der Arbeitsunfähigkeitstage innerhalb der letzten 6 Monate, M (SD)**	22 (33)	22 (32)	25 (40)	0,476	31 (43)	19 (36)	0,207
Fehlend, *n* (%)	1 (0)	1 (0)	0 (0)		0 (0)	0 (0)	
**Depression**^e^**, M (SD)**	13 (5,1)	13 (5,0)	14 (5,7)	0,224	14 (6,0)	13 (5,4)	0,418
Fehlend, *n* (%)	1 (0)	1 (0)	0 (0)		0 (0)	0 (0)	
**Ängstlichkeit**^f^**, M (SD)**	3,5 (1,7)	3,4 (1,7)	3,6 (1,6)	0,299	3,7 (1,6)	3,6 (1,6)	0,684
Fehlend, *n* (%)	1 (0)	1 (0)	0 (0)		0 (0)	0 (0)	
**Somatisierung**^g^**, M (SD)**	13 (5,8)	13 (5,6)	14 (6,7)	0,442	14 (6,9)	13 (6,6)	0,593
Fehlend	14 (3)	12 (2)	2 (3)		2 (6)	0 (0)	

^a^Migrationshintergrund

^b^Berechnet mit exakten Fisher-Tests, Boschloos Tests oder 2‑seitigen (Welch-)t-Tests, signifikante Unterschiede werden fett hervorgehoben

^c^Migrationsgeneration

^d^Prozentangabe gemessen an allen Personen mit Migrationshintergrund

^e^PHQ‑9 mit einem Wertebereich von 0 bis 3 pro Item

^f^GAD‑2 mit einem Wertebereich von 0 bis 3 pro Item

^g^SSS‑8 mit einem Wertebereich von 0 bis 4 pro Item

### Zusammenhänge zwischen allgemeinen soziodemografischen, migrations- und arbeitsbezogenen Daten und der mentalen Gesundheit

#### Gesamte Stichprobe.

Die Ergebnisse der multivariablen linearen Regressionsanalysen für die Gesamtstichprobe sind Tab. 4 zu entnehmen. Allgemeine soziodemografische, migrations- und arbeitsbezogene Angaben hingen nicht mit Depressivität zusammen (alle *p* ≥ 0,076, alle β ≤ |0,08|). Das weibliche Geschlecht war sowohl mit höherer Ängstlichkeit (*p* = 0,019, β = 0,11) als auch höherer Somatisierung (*p* < 0,001, β = 0,29) assoziiert. Ein höheres Alter (*p* < 0,001, β = 0,15) sowie Schichtarbeit mit Nachtschicht (im Vergleich zu einer Arbeit ohne Schichtarbeit) hingen mit einer höheren Somatisierung zusammen (*p* = 0,012, β = 0,11).

**Tab. 4:** Zusammenhänge zwischen sozioökonomischen, geschlechterspezifischen und migrationsbedingten Angaben und der mentalen Gesundheit (Depressivität, Ängstlichkeit, Somatisierung) in der gesamten Stichprobe

Unabhängige Variablen	Modell 1: Depressivität (PHQ-9)^a^ (R^2^ = 0,02, R^2^_adj_ = 0,0034)				Modell 2: Ängstlichkeit (GAD-2)^b^ (R^2^ = 0,017, R^2^_adj_ = 0,00)				Modell 3: Somatisierung (SSS-8)^c^ (R^2^ = 0,07, R^2^_adj_ = 0,06)			
	Regressionskoeffizient (95 % KI)	SE	Beta	*p*	Regressionskoeffizient (95 % KI)	SE	Beta	*p*	Regressionskoeffizient (95 % KI)	SE	Beta	*p*
Konstante	13,21	1,74	–	**<** **0,001**	3,85	0,58	–	**<** **0,001**	11,17	1,92	–	**<** **0,001**
Geschlecht^d^	0,82 (−0,08–1,72)	0,46	0,08	0,076	0,36 (0,08–0,66)	0,15	0,11	**0,019**	2,27 (1,27–3,27)	0,51	0,20	**<** **0,001**
Alter	−0,02 (−0,06–0,02)	0,02	−0,04	0,338	< 0,01 (−0,02–0,02)	0,01	−0,02	0,573	0,08 (0,04–0,12)	0,02	0,15	**<** **0,001**
Familienstand^e^	−0,13 (−0,05–0,79)	0,47	−0,01	0,788	−0,92 (−1,23–0,61)	0,16	−0,05	0,222	−0,61 (−1,63–0,41)	0,52	−0,05	0,241
Wöchentliche Arbeitsstunden	0,02 (−0,04–0,08)	0,03	0,02	0,596	< 0,01 (−0,02–0,02)	0,01	−0,02	0,706	−0,04 (−0,12–0,04)	0,04	−0,05	0,262
Schichtarbeit mit Nachtschicht^f^	1,39 (−0,28–3,06)	0,85	0,07	0,103	< 0,01 (−0,57–0,57)	0,29	< 0,01	0,100	2,40 (0,54–4,26)	0,95	0,11	**0,012**
Schichtarbeit ohne Nachtschicht^g^	1,45 (−0,37–3,27)	0,93	0,07	0,119	−0,12 (−0,73–0,49)	0,31	−0,02	0,695	1,56 (−0,50–3,62)	1,05	0,06	0,140
Führungsposition^h^	0,20 (−0,88–1,28)	0,55	0,02	0,719	0,05 (−0,32–0,42)	0,19	0,01	0,695	0,13 (−1,07–1,33)	0,61	0,01	0,833
MH^i^	−0,74 (−2,07–0,59)	0,68	−0,05	0,274	−0,21 (−0,66–0,24)	0,23	−0,04	0,345	−1,25 (−2,72–0,22)	0,75	−0,07	0,095

^a^5 Beobachtungen als fehlend gelöscht

^b^5 Beobachtungen als fehlend gelöscht

^c^18 Beobachtungen als fehlend gelöscht

^d^Weiblich vs. männlich/divers (Ref.)

^e^Ledig/geschieden/verwitwet vs. verheiratet/in fester Partnerschaft (Ref.)

^f^Schichtarbeit mit Nachtschicht vs. keine Schichtarbeit (Ref.)

^g^Schichtarbeit ohne Nachtschicht vs. keine Schichtarbeit (Ref.)

^h^Führungsposition Ja vs. Nein (Ref.)

^i^Migrationshintergrund nicht vorhanden vs. vorhanden (Ref.)

#### Personen mit Migrationshintergrund.

Die Ergebnisse der multivariablen linearen Regressionsanalysen für die Personen mit MH werden in Tab. 5 präsentiert. Ein marginal signifikanter Zusammenhang lässt sich zwischen dem weiblichen Geschlecht und Somatisierung erkennen (*p* = 0,053, β = 0,26). Ansonsten zeigten weder die übrigen allgemeinen soziodemografischen noch die migrations- oder arbeitsbezogenen Angaben einen Zusammenhang mit der mentalen Gesundheit (alle *p* ≥ 0,072, alle β ≤ |0,24|). Eine Orientierung an der Herkunftskultur zeigte jedoch tendenziell einen Zusammenhang mit geringerer Somatisierung (*p* = 0,084, β = −0,23), während eine Führungsposition die Tendenz aufwies, mit höherer Depression (*p* = 0,098, β = 0,22) und Somatisierung assoziiert zu sein (*p* = 0,072, β = 0,24).

**Tab. 5:** Zusammenhänge zwischen sozioökonomischen, geschlechterspezifischen und migrationsbedingten Angaben und der mentalen Gesundheit (Depressivität, Ängstlichkeit, Somatisierung) in der Subpopulation der Personen mit Migrationshintergrund (erste und zweite Migrationsgeneration)

Unabhängige Variablen	Modell 1: Depressivität (PHQ-9)^a^ (R^2^ = 0,14, R^2^_adj_ = 0,00)				Modell 2: Ängstlichkeit (GAD-2)^b^ (R^2^ = 0,11, R^2^_adj_ = −0,03)				Modell 3: Somatisierung (SSS-8)^c^ (R^2^ = 0,21, R^2^_adj_ = 0,08)			
	Regressionskoeffizient (95 % KI)	SE	Beta	*p*	Regressionskoeffizient (95 % KI)	SE	Beta	*p*	Regressionskoeffizient (95 % KI)	SE	Beta	*p*
Konstante	23,00	6,48	–	**<** **0,001**	4,64	1,93	–	**0,020**	20,03	7,34	–	**0,009**
Geschlecht^d^	0,41 (−2,59–3,41)	1,53	0,04	0,789	0,00 (−0,88–0,88)	0,45	< 0,01	0,998	3,35 (0,02–6,63)	1,70	0,26	0,053
Alter	−0,08 (−0,24–0,08)	0,08	−0,16	0,291	−0,04 (−0,08–0,00)	0,02	−0,23	0,141	−0,03 (−0,21–0,15)	0,09	−0,05	0,729
Orientierung an Aufnahmekultur	−0,15 (−0,33–0,03)	0,09	−0,22	0,119	−0,02 (−0,08–0,04)	0,03	−0,08	0,580	−0,08 (−0,23–0,14)	0,11	−0,10	0,471
Orientierung an Herkunftskultur	−0,03 (−0,13–0,07)	0,05	−0,08	0,582	0,00 (−0,04–0,04)	0,02	0,01	0,941	−0,11 (−0,23–0,01)	0,06	−0,23	0,084
Wöchentliche Arbeitsstunden	0,04 (−0,16–0,24)	0,10	0,06	0,670	0,03 (−0,03–0,09)	0,03	0,16	0,247	−0,01 (−0,23–0,21)	0,11	−0,02	0,906
Schichtarbeit mit Nachtschicht^e^	3,84 (−1,71–9,39)	2,83	0,19	0,181	0,33 (−1,32–1,98)	0,84	0,06	0,700	4,51 (−1,64–10,66)	3,14	0,19	0,156
Schichtarbeit ohne Nachtschicht^f^	1,41 (−3,59–6,41)	2,55	0,08	0,582	−0,81 (−2,30–0,68)	0,76	−0,15	0,293	0,49 (−6,00–6,98)	3,31	0,02	0,882
Führungsposition^g^	3,89 (−0,64–8,42)	2,31	0,22	0,098	0,58 (0,36–0,80)	0,69	0,11	0,404	4,74 (−0,32–9,80)	2,58	0,24	0,072

^a^6 Beobachtungen als fehlend gelöscht

^b^6 Beobachtungen als fehlend gelöscht

^c^8 Beobachtungen als fehlend gelöscht

^d^Weiblich vs. männlich/divers (Ref.)

^e^Schichtarbeit mit Nachtschicht vs. keine Schichtarbeit (Ref.)

^f^Schichtarbeit ohne Nachtschicht vs. keine Schichtarbeit (Ref.)

^g^Führungsposition Ja vs. Nein (Ref.)

## Diskussion

Unter Berücksichtigung sozioökonomischer, geschlechter- und migrationsbedingter Merkmale wurde in der vorliegenden Arbeit explorativ der Zusammenhang eines möglichst breiten Spektrums unterschiedlicher Risikofaktoren auf die psychische Gesundheit in Form von Depressivität, Ängstlichkeit und Somatisierung an psychisch belasteten Beschäftigten in Deutschland geprüft. Frauen zeigten höhere Angst- und Somatisierungsausprägungen als Männer. Höheres Alter sowie Nachtschichtarbeit waren mit höheren Somatisierungssymptomen assoziiert. Familienstand, wöchentliche Arbeitsstunden, Schichtarbeit ohne Nachtschicht, das Innehaben einer Führungsposition und der MH zeigten keinerlei Zusammenhang mit der mentalen Gesundheit für die gesamte Stichprobe. Bei Betrachtung der Personen mit MH (erste und zweite MG) konnten keine signifikanten Zusammenhänge, jedoch statistische Tendenzen festgestellt werden. Auch hier war ein weibliches Geschlecht tendenziell mit höheren Somatisierungssymptomen assoziiert. Das Innehaben einer Führungsposition ging tendenziell mit einer höheren Depressivität und Somatisierung einher. Eine Orientierung an der Herkunftskultur war tendenziell mit geringerer Somatisierung assoziiert. Alter, Familienstand, wöchentliche Arbeitsstunden sowie Arbeit im Schichtdienst mit oder ohne Nachtschicht zeigten keinen Zusammenhang mit der mentalen Gesundheit unter Personen mit MH.

Die Ergebnisse zum Zusammenhang mit der allgemeinen Soziodemografie stehen teilweise im Einklang mit Befunden vorangegangener Forschung. Das Alter zeigte auch in anderen Studien einen signifikanten Zusammenhang mit höherer Somatisierung bzw. körperlichen Schmerzsymptomen [24, 25]. Dies liegt daran, dass ältere Personen häufiger von körperlichen Erkrankungen betroffen sind. Frauen litten auch in vorangehenden Studien häufiger unter einer schlechteren psychischen Gesundheit als Männer [25–27]. Dies könnte darin begründet sein, dass Frauen weltweit systematisch benachteiligt werden. In den meisten Gesellschaften besitzen sie einen niedrigeren Status und weniger Kontrolle über die Entscheidungsfindung in Bezug auf ihren Körper, ihre Beziehungen, Familien und Gemeinschaften als Männer, wodurch sie häufiger Gewalt und Zwang ausgesetzt sind [28], was sich auf die psychische Gesundheit niederschlagen könnte. Zudem begünstigen biologische Faktoren wie genetische und hormonelle Merkmale die Entwicklung psychischer Symptome bei Frauen [29]. Der Zusammenhang zwischen Nachtschichtarbeit und Somatisierung wurde ebenso bereits bestätigt [30]. Ein Grund hierfür wird in der Störung des inneren zirkadianen Rhythmus und einer damit einhergehenden unzureichenden Erholung aufgrund von Schlafstörungen vermutet [31].

Weder für Depressivität noch für Ängstlichkeit spielte der MH eine bedeutsame Rolle. Im Gegensatz dazu lässt vorherige Forschung darauf schließen, dass (türkische) Migrant:innen im Vergleich zu Personen ohne MH erhöhte Prävalenzraten für Depressivität und Somatisierung aufwiesen [12]. Ein Grund für den fehlenden signifikanten Effekt des MH könnte in der zu geringen Teststärke liegen, die aufgrund der relativ kleinen Stichprobe der Personen mit MH (*n* = 68) sowie der sehr unterschiedlich großen Subgruppen anzunehmen ist. Einen Hinweis liefern die auch bei nicht signifikanten Unterschieden teilweise hoch ausfallenden standardisierten Regressionskoeffizienten (β-Gewichte). Somit war die Stichprobe der Personen mit MH vermutlich nicht groß genug, um einen bedeutsamen Effekt aufdecken zu können. Dies wird dadurch bestärkt, dass eine deskriptive Betrachtung der Mittelwerte aller Gesundheitsoutcomes, v. a. in Bezug auf Somatisierung, zeigt, dass Personen mit MH die Tendenz aufwiesen, stärker belastet zu sein als Personen ohne MH.

Die Ergebnisse deuten darauf hin, dass in die vorliegende Stichprobe insbesondere die Arbeitnehmer:innen mit MH eingeschlossen wurden, die bereits sowohl sozial, sprachlich als auch ökonomisch sehr gut integriert waren. Dies lässt sich an der relativ langen Aufenthaltsdauer (24 Jahre) der Personen der ersten MG in Deutschland und ihren guten Deutschkenntnissen (33 % sehr gut oder gut) erkennen und könnte dazu geführt haben, dass die hier untersuchten Personen mit MH über eine bessere mentale Gesundheit verfügten als die Gesamtheit der Personen mit MH in Deutschland. Dass die hier untersuchten Personen mit MH bereits sehr gut integriert sein könnten, könnte auch dafür verantwortlich sein, dass der Grad der soziokulturellen Akkulturation (FRAKK) sowie die Belastung bezüglich Migrationsanforderungen (DIS) keine signifikanten Zusammenhänge mit der mentalen Gesundheit aufwiesen, obwohl dies in vorangegangener Forschung nachgewiesen wurde [32, 33]. Dies könnte darin begründet liegen, dass sich die hier befragten Personen mit MH bereits stark an der Aufnahmekultur orientierten und keine belastenden Anforderungen in Bezug auf ihre Einwanderung mehr wahrnahmen. Diese Annahme wird durch die vorliegenden deutlich höheren Werte in der Orientierung an der Aufnahmekultur als in der Orientierung an der Herkunftskultur sowie die geringe Belastung durch Migrationsanforderungen untermauert.

Dass unter den Personen mit MH das Bekleiden einer Führungsposition tendenziell mit höherer Depression sowie Somatisierung einherging, lässt sich dadurch begründen, dass Führungspersonen mit MH u. U. häufiger die Erfahrung machen, aufgrund ihres MH als Autoritätspersonen nicht respektiert zu werden. Im Einklang damit wurde bereits gezeigt, dass auch migrantische Personen mit einem hohen Bildungsstand von Diskriminierung betroffen sind [34]. Diese Erfahrung könnte bei Personen mit MH in Führungspositionen mit einer erhöhten Stressbelastung und somit höherem Risiko für mentale Gesundheitsprobleme einhergehen.

Insgesamt bestätigen die Ergebnisse, dass die Stichprobe der psychisch belasteten Beschäftigten in der vorliegenden Arbeit sowohl Symptome klinisch relevanter Depressivität (M = 13,0, SD = 5,1), Ängstlichkeit (M = 3,5, SD = 1,7) sowie Somatisierung (M = 13,0, SD = 5,8) aufwies. Bezüglich aller 3 Aspekte der mentalen Gesundheit erreichten die hier befragten Beschäftigten einen höheren Wert als die Allgemeinbevölkerung in Deutschland (Depression (PHQ-9): M = 3,30, SD = 3,65 [35], Ängstlichkeit (GAD-2): M = 1,12, SD = 1,39 [36], Somatisierung (SSS-8): M = 3,23, SD = 3,96 [22]). Dies spiegelt sich auch in der durchschnittlichen Anzahl der Arbeitsunfähigkeitstage innerhalb der letzten 6 Monate wider, die mit 22 Tagen weit über dem Durchschnitt in der Allgemeinbevölkerung mit 15 Tagen [37] liegt. Da die vorliegende Stichprobe aber aus Beschäftigten bestand, die sich als psychisch belastet empfanden, waren diese Ergebnisse nicht überraschend.

### Stärken und Schwächen der Studie

Eine Stärke besteht darin, dass im Rahmen dieser Studie Beschäftigte an 5 verschiedenen Standorten in Deutschland rekrutiert wurden und so mögliche Verzerrungen aufgrund lokaler sozioökonomischer, arbeitsbezogener oder gesellschaftlicher Gegebenheiten vermindert wurden. Eine weitere Stärke liegt in der aufwendigen Schulung der Betriebsärzt:innen, über die die Proband:innen rekrutiert wurden. Diese Schulungen beinhalteten die Sensibilisierung der Rekrutierenden bezüglich Personen mit MH, deren Krankheitskonzepte und Bedürfnisse sowie die interkulturelle Kommunikation. Das Ziel hierbei bestand darin, Personen mit MH dem Bevölkerungsanteil entsprechend für die Teilnahme an der Studie gewinnen zu können. Zudem ist die Erfassung der Personen mit MH nach der Definition des Mikrozensus [17] als Stärke zu nennen, da diese häufig in der epidemiologischen Migrationsforschung verwendet wird, was eine gute Vergleichbarkeit zwischen Studien zu Personen mit MH in Deutschland ermöglicht. Eine weitere Stärke der vorliegenden Studie kann in der Vielzahl an arbeits- sowie migrationsspezifischen (z. B. Akkulturation, postmigratorische Belastungen) erhobenen Merkmalen gesehen werden, welche eine facettenreiche Analyse ermöglichte.

Eine Limitation stellt die Verwendung von Selbstbeurteilungsfragebögen dar, da sie aufgrund von sozialer Erwünschtheit anfällig für Verzerrungen sind [38]. Zudem erlaubt das Querschnittdesign keine Rückschlüsse auf kausale Zusammenhänge, was jedoch zusätzliche Erhebungszeitpunkte ermöglichen werden. Ein Blick auf die Determinationskoeffizienten der Regressionsanalysen legt außerdem sehr niedrige R^2^-Werte offen. Dies ist ein Indiz dafür, dass wichtige, mit den Gesundheitsoutcomes zusammenhängende Faktoren im Rahmen der Studie nicht erhoben oder nicht in die Analysen einbezogen wurden. Zukünftige Studien sollten demnach weitere sozioökonomische (z. B. Bildungsgrad), arbeits- (z. B. spezifische Arbeitsbedingungen) und migrationsspezifische Faktoren (z. B. Geflüchtetenstatus) berücksichtigen.

Auch darf die doppelte Vorselektion der Teilnehmenden nicht außer Acht gelassen werden. Einerseits wurde hauptsächlich in Betrieben, die einer Kooperation zugestimmt haben, rekrutiert. Jedoch wurde durch die Rekrutierung über soziale Medien und Zeitungsartikel versucht, diesem Selektionsbias entgegenzuwirken. Zudem handelt es sich bei den Proband:innen um Personen, die sich selbst als psychisch belastet wahrnahmen und für sich das Bedürfnis einer frühen Intervention am Arbeitsplatz sahen. Aus diesen Gründen muss bei der Interpretation der Ergebnisse bedacht werden, dass diese nicht ausnahmslos auf die Gesamtheit der Beschäftigten in Deutschland generalisiert werden können. Dies zeigt sich auch in der Verteilung soziodemografischer sowie arbeitsbezogener Faktoren im Vergleich zu Beschäftigten in der deutschen Allgemeinbevölkerung. In einigen Merkmalen finden sich ähnliche Verteilungen (z. B. Alter: 46 Jahre vs. 44 Jahre [39], wöchentliche Arbeitsstunden: 37 h vs. 35 h [40], Schichtarbeit: 13 % vs. 16 % [41]), bezüglich anderer Charakteristika Unterschiede (Frauenanteil: 55 % vs. 47 % [42], Anstellung in Großunternehmen: 80 % vs. 45 % [43]). Dennoch liefert die vorliegende Studie auf explorative Art wichtige Hinweise für zukünftige Studien.

Eine weitere Einschränkung besteht im Gruppengrößenunterschied zwischen Personen mit und ohne MH. Eine Ursache hierfür liegt darin, dass Personen mit MH meist schwer zu erreichen sind („hard to reach population“; [44]), da ihre Bereitschaft, Fragebögen zu beantworten, als geringer gilt als unter Personen ohne MH [45–47], z. B. aufgrund von Sprachbarrieren [47]. Aus statistischer Sicht könnte dies dazu geführt haben, dass die Teststärke nicht groß genug war, um kleine, aber aussagekräftige Effekte aufzudecken. Weiterhin zeigt sich eine Unterrepräsentanz von Personen mit MH im Vergleich zur deutschlandweiten Verteilung. Im Jahr 2021 besaßen unter insgesamt 33,8 Mio. Beschäftigten in Deutschland 31 % einen MH [48]. In der vorliegenden Studie betraf dies jedoch nur 12 %. In zukünftigen Studien sollte der sog. Community-Ansatz verfolgt werden, um auch die weniger gut integrierten Personen mit MH zu rekrutieren. Hierbei werden Schlüsselpersonen der entsprechenden Subpopulation aufgesucht, um diese für die Teilnahme an wissenschaftlichen Arbeiten zu motivieren [49]. Auch der Einsatz muttersprachlicher Studienteammitglieder könnte eine höhere Teilnahmemotivation unter Personen mit MH begünstigen.

## Fazit

Die vorliegende Untersuchung verdeutlicht die hohe psychische Belastung der teilnehmenden Beschäftigten und damit die hohe Relevanz von Interventionsangeboten, die auf vulnerable Gruppen zugeschnittenen sind. Um psychischen Belastungen entgegenzuwirken, die sich in psychischen Störungsbildern und folglich Arbeitsunfähigkeitstagen und damit einhergehenden hohen Kosten im Wirtschaftssystem manifestieren können, sollten Arbeitgeber:innen für psychische Leiden ihrer Arbeitnehmer:innen sensibilisiert werden. Auf politischer Ebene sowie der Ebene der Arbeitgeber:innen sollten Unterstützungsangebote für Beschäftigte wie leicht zugängliche frühe psychotherapeutische Maßnahmen angeboten und deren Inanspruchnahme unterstützt und gefördert werden. Besonderes Augenmerk sollte dabei auf Gruppen gelegt werden, die besonders von psychischen Belastungen betroffen sind: Frauen, ältere Mitarbeitende und Beschäftigte mit Arbeitsbedingungen, die sich ungünstig auf die psychische Gesundheit auswirken, wie etwa Nachtschichtarbeit. Zur Verbesserung des psychischen Gesundheitszustands von Schichtarbeiter:innen in der Nachtschicht sollten Maßnahmen eingeführt werden, die Strategien zur Verbesserung der Schlafqualität umfassen und sowohl Änderungen des individuellen Verhaltens und der Schlafumgebung als auch eine Umgestaltung der Arbeitszeiten auf Unternehmensebene beinhalten.
